# Four Reports Summitted on July 15, 1917, at a Meeting of the Chiefs of the Centers of Treatment for Skin and Venereal Diseases

**Published:** 1917-11

**Authors:** 


### SKIN AND GENITO-URINARY

Four Reports Summitted on July 15, 1917, at a Meeting of the Chiefs of the Centers of Treatment for Skin and Venereal Diseases. Translated and condensed from the *Archives de Médecine et de Pharmacie Militaires*, May, 1917.

I. — Concerning the Best Distribution of Skin and Venerial Centers. By M. O. Pasteau, Chief of a genito-urinary Center of the Army.

I. — It is better to have one important center for each army than several lesser ones:

1. It gives unity to the treatment and facilitates medical records.

2. It makes possible considerable economy in specialists.

3. It avoids delay in transportation and in the return to service after treatment.

4. It permits close observation with a view to relapse.

5. It may be at the same base as the dispensary for the treatment of the infected women of the region.

II. — Conditions desirable in such a center.

*a.* A well closed-in establishment, sufficiently large to accommodate easily about 600 to 800 patients for a period of about a month each. The conditions for rest should be satisfactory. The formation ought not to be so far back as to cause delay when the patient is ready to return to service, or so situated as to offer temptations for further contamination. It ought not to be too far forward, however, lest the conditions prevent proper treatment and speedy recovery.

*b.* The center for skin and venereal diseases *should remain an integral part of the army organization, under the direction of the army physician, and should be situated within the army zone.* It should never become a regional formation. Its proper place is within the zone of the army's movements. Light cases (like scabies and urethritis without complications) need not be removed to the center for treatment, but may be handled in the camp infirmaries.

*c.* The center ought to be situated on some important railway line in the rear of the army, along which the troops regularly pass.

*d.* It is best situated in a railway center, or, if that would be too remote, it may be placed along the main line at about 50 km. from the front lines. Thus it may be constantly and easily accessible, whatever the fortunes of the war.

II. — PROSTITUTION IN THE CANTONMENTS AND IN THE NEIGHBORHOOD OF THE CAMPS: ITS SURVEILLANCE AND SUPPRESSION. By M. Spillmann, Major and Chief of the Venereological Center of the 22nd District.

Increase in the number of venereal cases is in proportion to the increase in prostitution.

I. *Cantonments and Camps.* The camps chiefly concerned are those for instruction with fixed establishments and those for exercise or preparation for combat which are often situated among scattered villages. These amount to an assemblage of cantonments.

II. *What Women are Prone to Prostitution in these Various Regions?*

1. There are none in cantonments immediately behind the lines.

2. Rest cantonments at a short distance, however, are usually beset with women who are there expressly for this purpose. They are generally sole occupants of a house, and may practice prostitution secretly.

3. In cantonments for complete rest, situated in normal towns and villages, the number of women of this character may be the same as in peace times. The conduct of many of them should be watched.

III. *What Should Our Attitude Be towards Prostitution?*

In the course of a long war, prostitution is not at all the same as in peace times. The field of prostitution is at present greatly enlarged; of this professional prostitution constitutes only a small part. Married women are in large numbers afflicted with disease, and their surveillance demands great tact and discretion. There is, therefore, a great deal of clandestine prostitution, which, because of present legislation, can not be watched. This type is by far the most dangerous.

It is highly desirable to draw up a list of as many prostitutes as possible, and to watch and care for those who are known to be diseased.

IV. *How May Such a List Be Drawn up? What Is Being Done? What Ought to Be Done?*

When a detachment arrives at a cantonment, the Commander should try at once to learn who the prostitutes are, so as to make sure that they are properly watched. An agent of the police force should carry out the actual enrollment. In all cases the army authorities should be kept in touch with all stages of the proceedings, and they should act in complete accord with the civil authorities.

V. *What Results are Obtained by Inscription.*

In some villages no list of prostitution exists. In others there is a list, but not of the women themselves. When the names of women are given, they are usually but a small part of the profession. Indeed, there remedial means are of slight value when compared with the evil as a whole.

VI. *Women under Surveillance.*

In certain regions at the front, in an attempt to combat venereal disease more effectively, the authorities have arbitrarily drawn up a list of women, not already registered, but, who, it appears, should be carefully but delicately watched. This surveillance is carried out under the military and municipal authority. Every woman so noted should be informed that she will be enrolled upon the public list, unless she presents each week to the authorities a medical certificate. The certificate should be issued gratuitously by a specially appointed physician. This method of procedure is strongly recommended.

VII. *Surveillance of Prostitution.*

If there are factories employing women in the neighborhood of the cantonments, the troops should not be allowed to visit these establishments more than is necessary.

Inns, cafés, and hotels should be rigidly watched.

A card of identity should be demanded for any woman entering a chamber with a soldier or officer (as formally prescribed by the Minister of the Interior, May 30, 1917).

Such regulations have no doubt given good results, but it is possible to err on the side of too much strictness. The ruling of the Minister referred to, also provides that proscribed women shall not be allowed to approach the railway stations, barracks, places of instruction, squares, markets, public promenades, etc. The result of such rigid confinement is that recognized prostitutes are replaced by secret ones.

Nevertheless there should be a surveillance of all places that might serve as *rendez-vous.* The surveillance, however, should be chiefly for the purpose of hunting out uninscribed women and examining them. *Regulated prostitution should be protected and not harrassed.*

The physician charged with the inspection of the listed prostitutes or those in houses, ought not to employ feed delegates in the performance of his duties, for fear of losing his independence.

In the case of clandestine prostitutes, the task is difficult, and surveillance is hardly possible, except as a result of information given by the man contaminated.

When such a woman is submitted to examination, the doctor should :

1. Instruct the woman as to the possible consequences of the disease which she may spread.

2. Induce her to submit to necessary treatment, even if this involves hospital care.

Qualified doctors should be appointed by the military authorities to control the work done by the police in the search for such women : but the physician ought never to encroach upon the prerogatives of the police, nor should the police interfere in matters strictly medical.

Antivenereal education should also be actively carried on for the men in the service. In this way the individual sanitary card issued to the woman by the doctor may render an important service.

VIII. *Is It Possible to Make the Inspection More Effective by Constraint?*

The usual constraint practiced by the municipal and prefectoral authorities is inadequate. The present system of fine or imprisonment is not effective.

In the war zones the woman should be expelled after two failures to appear for examination; or, better yet, sent to a hospital, since such a woman is almost invariably diseased.

IX. *The Hospital Treatment of the Women.*

Women should come for treatment confidentially. They ought to come of their own accord as soon as they find themselves diseased. The recent circular issued by the Minister of the Interior on the treatment of venereal diseases is, in this particular, a marked step in advance. If the first and most important measure is the surveillance of as many women as possible, the second is to increase as much as possible the number of those who voluntarily submit to treatment.

It is best, therefore, not to persecute uselessly the women of the profession, but to facilitate the approach of medical assistance to diseased women, and, above all, to encourage women so afflicted to come voluntarily for treatment before they are constrained to do so by force.

*Conclusions* :

The author reviews the points made above and adds : Every woman enrolled should be examined medically twice a week. No person on the list should be allowed to change her residence without first satisfying the sanitary authorities that she is not diseased. Women procurers should especially be watched. Women who live in the neighborhood should never be allowed access to the camps. A circle of at least one and a half kilometer in breadth should be established about each camp, and carefully guarded against such women. Provision should be made for defraying the expenses of syphilitic cases, and for the maintainance of the children whose mothers are obliged to undergo hospital treatment.

III. Organization of Supplementary Services. Difficulties Encountered. — By M. Gougerot, Chief of the Dermatological Center of the IXth District.

Supplementary services constitute one of the most powerful means of combating venereal disease, and at present they are the most easily established. They should be multiplied and fully developed[1].

A supplementary service should accomplish five objects :

1. The treatment of civilians with venereal diseases and women *who are not prostitutes*. Irregular consultations and treatment should be arranged for, outside of working hours; necessary hospital treatment should be provided; there should be a laboratory to determine the diagnosis in doubtful cases; and provision for the careful supervision of treatment and of the pronouncement of cures.

2. The surveillance of prostitutes by means of periodical examinations, irregularly arranged consultations and treatment free of charge, and internment in hospitals other than those used for patients who are not prostitutes.

3. Measures against secret prostitution.

4. Discovery of the centers of contagion.

5. Propaganda, lectures, pamphlets, etc..., and prophylactic education in general.

The chief of the supplementary service ought to centralize the venereal work of his section.

Unfortunately these arrangements meet with numerous obstacles. They are here enumerated with suggestions for overcoming them.

I. *Objections on Principle* :

The author considers the various reasons advanced in towns by the civil authorities against these services. It is often argued that such free dispensaries establish a bad precedent, and will

1. The IXth district has been provided with eleven supplementary services, and the system is complete except for Tours, where the municipality refused to permit one.

ultimately necessitate treatment of other common complaints at the public expense. It is also contended that no one will come voluntarily to such places because by so doing they proclaim their guilt.

The writer answers these objections by insisting upon the advantages to be derived by the community from the advanced scientific methods employed in such services as against the older forms of treatment, and from the arrangement for treatment and consultation outside of working hours, thus preventing the loss to the laborer or his employer that resulted from the old methods of treatment during working hours. Furthermore, the writer declares that financially the town physicians and druggists have little to fear, since the patients who would profit by the free treatment are for the most part from the poorest class. To avoid the fear of publicity on the part of the patient, it is well to have all the sick of the town come for consultation at the same time, and then venereal cases may be segregated without arousing suspicion.

II. *Objections of an Administrative Order.*

Under this heading the author considers such difficulties as the centralizing of authority when a military establishment is situated in a civilian district, the cost of installation, the dearth of buildings, the lack of properly trained specialists, the difficulty of unifying methods of treatment, cooperation with dental service in cases of serious mercurial treatment, arrangement for proper bacteriological control, increased cost of medicines, local prejudices, and cautious publicity for the work. The writer believes that all these obstacles may and should be overcome. He also recommends an ambulance service by which doctors may visit laboring centers at some distance from the supplementary service hospital, often making use of a factory infirmary for the actual inspection and treatment.

IV. ORGANIZATION OF THE SURVEILLANCE EXERCISED WITH REGARD TO VENEREAL DISEASES IN WAR FACTORIES. By M. J. Nicolas, Major, and Professor and Specialist in Skin and Venereal Diseases on the Faculty of Medicine at Lyon.

I. *The Part Played by War Factories in the Propagation of Venereal Diseases.*

War factories are not simply places where such diseases are commonly observed, but the conditions under which men and women labor in them with utmost promiscuity, and with foreigners and colonials added, make them veritable homes for the propagation of such maladies.

II. *A Study of the Measures Taken to Check the Propagation of Venereal Disease in Factories.*

A. *Systematic Surveillance.*

This involves the careful personal inspection of all those employed in the factory by a competent venereal specialist at least once, or, better, twice each month. Theoretically this is the ideal method, but it is impracticable for physiological, moral, and material reasons.

1. Both men and women would resent and absolutely refuse to submit to such investigations as unlawful encroachments upon their liberty. Nevertheless, working women whose conduct is highly offensive, ought to be subjected to medical inspection while at work, and should be obliged to submit to treatment, in a hospital if need be, until the cure is complete. Article 4 of the notification to prefects from the Minister of the Interior gives authorization to this effect.

2. Still more serious is the difficulty of adequately examining such vast numbers of workers. At Lyon there are 111,500, and in the XIV[th] district there are from 250,000 to 300,000. To inspect such a number twice a month — and to do so less frequently would be of little avail — 60 physicians at least, doing nothing else, would be required.

3. The directors of the factories would object seriously to having the inspection during work hours, and the laborers still more seriously to having their rest or meal time invaded in such a fashion. This latter method, too, would require an incalculable number of doctors, as the laborers usually live at a considerable distance from their factories, and would have to be visited by special arrangement.

Except, then, for a limited number of the workers who can present no certificate of honest living, and for those who are notorious prostitutes, the method of systematic surveillance is out of the question.

B. *Medical, Educational, and Therapeutic Method.*

This means recourse to various procedures, the careful organization and coordination of which may lead to practical results, — if not perfect, at least of considerable importance.

*a.* Utilization of Ordinary Medical Visits for the Discovery of Venereal Infections.

Every person, man or woman, who comes to the factory physician on any pretext, should be examined carefully for symptoms of venereal disease. This doctor will then either proceed himself to administer the necessary treatment, or, better yet, refer the case to the nearest center for skin and venereal diseases.

*b.* Education of the Personel of the Factories concerning the Dangers of Venereal Sickness and the Necessity of Proper Treatment.

It is clear that the preceding method can be only superficial. The workers should therefore be instructed thoroughly in such matters, and persuaded to present themselves upon the slightest suspicion of infection, and without false modesty, either to the factory physician or else to the specialist at the center for skin and venereal diseases.

Professor Nicolas has required to be posted in all the war factories of the XIV$^{th}$ District, a placard which serves this purpose.

It may be well to go even further, and draw up more explicit notices such as have already been posted with regard to alcoholism and tuberculosis. Professor Nicolas has already prepared the text for such a notice.

*c.* The Multiplication of Dispensaries for Consultation and Supplementary Services for Dermato-Venereal Diseases.

These establishments should be made easily accessible for all. Outside of the large cities every important community, whether of factory workers or other civilians, should be provided either with a supplementary service, or at least, with means for consultation and treatment by appointment.

When the community of workers is too small for such special provision, at least every facility should be afforded the diseased (even to the expense of the journey) to obtain the necessary treatment. The most effective treatments are always employed. Medicines are furnished free of charge and without formality to all who demand them.

*d.* The Necessity of Informing Patients as to Thorough Treatment for Syphilis.

Syphilitics should be made to understand that once the symptoms of the disease have disappeared, they are not necessarily cured, and they should have it impressed upon them that to avoid a disagreeable return of the disease, they must submit to continued treatment administered at intervals and systematically. Such treatment may be carried on by the factory physicians themselves, or at least, superintended by them.

For this purpose, as well as for the treatment of simple urethritis, professor Nicolas has drawn up instructions to be distributed to all doctors stationed at depots or factories of war in the XIV$^{th}$ District. His example should be generally followed.

*Conclusions*,

After reviewing the points made above, the writer adds that in any case education is better than coercion. He recognizes, however, that in the case of foreigners and colonials education is not always possible. He believes that such laborers should be subjected to examination when it is possible to carry it out, but that this should be left to the decision of the heads of the divisional centers.

